# High-throughput next-generation sequencing for identifying pathogens during early-stage post-lung transplantation

**DOI:** 10.1186/s12890-021-01723-z

**Published:** 2021-11-07

**Authors:** Qiao-yan Lian, Ao Chen, Jian-heng Zhang, Wei-jie Guan, Xin Xu, Bing Wei, Dan-xia Huang, Jian-xing He, Chun-rong Ju

**Affiliations:** State Key Laboratory of Respiratory Disease, National Clinical Research Center for Respiratory Disease, Guangzhou Institute of Respiratory Health, The First Affiliated Hospital of Guangzhou Medical University, Guangzhou, China

**Keywords:** Lung transplantation, High-throughput next-generation sequencing, Pathogen, Pulmonary infection

## Abstract

**Background:**

High-throughput next-generation sequencing (HT-NGS) has the potential to detect a large variety of pathogens; however, the application of HT-NGS in lung transplant (LTx) recipients remains limited. We aimed to evaluate the value of HT-NGS for pathogen detection and diagnosis of pulmonary infection during early-stage post-lung transplantation.

**Methods:**

In this retrospective study, we enrolled 51 LTx recipients who underwent lung transplantation between January 2020 and December 2020. Bronchoalveolar lavage fluid (BALF) samples were collected for the detection of pathogens using both HT-NGS and conventional microbiological testing. The detection of pathogens and diagnostic performance of HT-NGS were compared with that of conventional methods.

**Results:**

HT-NGS provided a higher positive rate of pathogen detection than conventional microbiological testing (88.24% vs. 76.47%). The most common bacteria detected via HT-NGS during early-stage post-lung transplantation were *Enterococcus*, *Staphylococcus*, *Pseudomonas* and *Klebsiella*, while all fungi were *Candida* and all viruses were *Herpesvirus*. Uncommon pathogens, including *Strongyloides*, *Legionella*, and *Mycobacterium abscesses* were identified by HT-NGS. The sensitivity of HT-NGS for diagnosing pulmonary infection was significantly higher than that of conventional microbiological testing (97.14% vs. 68.57%; *P* < 0.001). For three LTx recipients, treatment regimens were adjusted according to the results of HT-NGS, leading to a complete recovery.

**Conclusion:**

HT-NGS is a highly sensitive technique for pathogen detection, which may provide diagnostic advantages, especially in LTx recipients, contributing to the optimization of treatment regimens against pulmonary infection during early-stage post-lung transplantation.

**Supplementary Information:**

The online version contains supplementary material available at 10.1186/s12890-021-01723-z.

## Background

Pulmonary infection is a severe complication with high morbidity and mortality among lung transplant (LTx) recipients [1]. LTx recipients are particularly prone to developing pulmonary infection and progressing rapidly due to the maintenance use of high-dose immunosuppressants, constantly exposed to various pathogens in environment, impaired cough reflex, the decreased mucociliary clearance, and etc.[2, 3]. Furthermore, LTx recipients are especially at a high risk of opportunistic infections and mixed infections [2, 4]. Therefore, accurate and timely etiological diagnosis of the infection is vital to enable appropriate treatment and improve prognosis, especially among LTx recipients who may need combination therapy for coinfection.

Conventional microbiological testing is commonly used in clinical settings, including smears, microbial cultures, serological tests, antigen/antibody assays, and polymerase chain reaction (PCR)-based nucleic acid detection [5, 6]. Although these methods have improved the detection rate of common pathogens, they can only detect pathogens within a specific range [7]. Clinical diagnosis continues to be remained difficult because of the low diagnostic yield of conventional microbiological testing, particularly in LTx recipients during the perioperative period, who are challenged by a complex pathogen spectrum [2, 8].

High-throughput next-generation sequencing (HT-NGS) is a new pathogen detection technology that allows thousands to billions of DNA fragments to be sequenced simultaneously and independently, enabling a fast and high positive rate for pathogen identification [9, 10]. Unbiased HT-NGS has been widely used to detect different types of pathogens, including bacteria, viruses, fungi, atypical pathogens, parasites and novel pathogens in clinical infectious diseases in recent years [11–15]. However, research exploring the application of HT-NGS in LTx recipients remains limited. To the best of our knowledge, only two articles have been reported, one is focused on the identification of viral species in LTx recipients, the other is about donor lung-colonized bacteria [16, 17]. The evaluation of HT-NGS for pathogen detection in LTx recipients is urgently needed because of the complex conditions and difficult management during the early period post-lung transplantation.

In this study, we analyzed the pathogens detected by HT-NGS in LTx recipients at early-stage post-lung transplantation, and aimed to elucidate the clinical impact of HT-NGS in pulmonary infection diagnosis by comparing the diagnostic yield between HT-NGS and conventional microbiological testing.

## Materials and methods

### Study population and study design

**Fig. 1:**
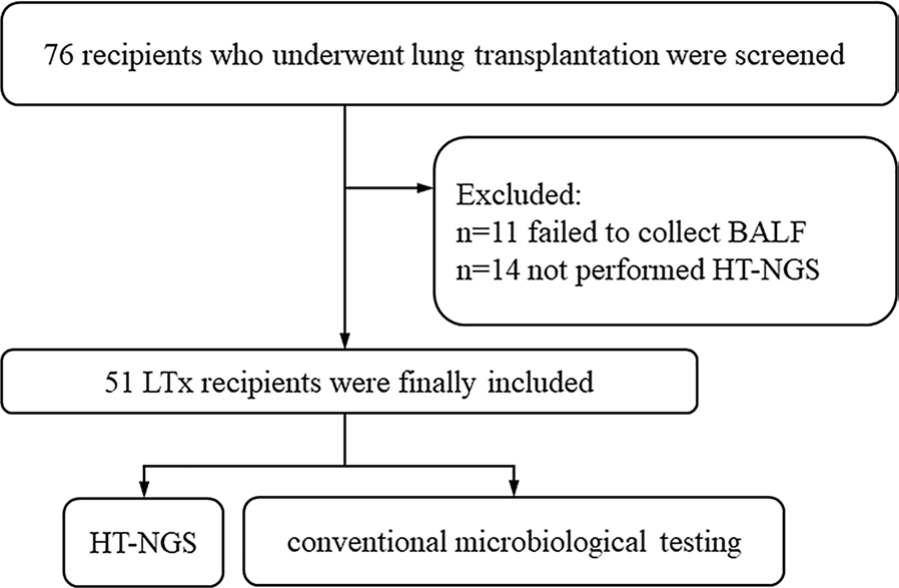
Flow diagram of the study

A total of 76 patients with end-stage lung disease underwent lung transplantation at the First Affiliated Hospital of Guangzhou Medical University from January 1 to December 31, 2020. In this single-center retrospective study, we enrolled 51 LTx recipients who underwent bronchoscopy test for bronchoalveolar lavage fluid (BALF) collection and consented to have their BLAF detected by HT-NGS during early-stage post-lung transplantation (Fig. 1).

Patients’ medical records were reviewed to collect baseline information, including demographic data, primary diseases before lung transplantation, type of surgery (unilateral, bilateral, or heart–lung transplantation), immunosuppressive regimen, conventional microbiological testing results, and the patients’ treatment regimen.

### Fiberoptic bronchoscopy, sample collection, and conventional microbiological testing

BALF samples were collected by experienced bronchoscopists using standardized procedures, from the lobes of the transplanted lung with the most prominent lesions according to chest computed tomography (CT) images. The lower lobe of the transplanted lung in LTx recipients were selected for lavage if lung lesions were not significant. Normal saline (50–60 mL) was injected at a targeted recovery rate of 40–60%. The BALF samples were immediately stored in sterilized containers and sent to the clinical laboratory, First Affiliated Hospital of Guangzhou Medical University for conventional microbiological testing, including smear and culture of bacteria, fungi, and mycobacteria; polymerase chain reaction (PCR) for cytomegaloviruses (CMV); galactomannan (GM) tests; *Mycobacterium tuberculosis* DNA testing; and Xpert MTB/RIF assay, within 2 h at room temperature (about 25 °C).

### High-throughput next-generation sequencing

#### Sample processing, sequencing, and data analysis

The BALF samples were transferred to a designated central laboratory for HT-NGS within 4 h and stored at 4 °C. DNA was extracted using the TIANamp Magnetic DNA Kit (Tiangen Biotech Co., Ltd., Beijing, China) according to the manufacturer’s protocol. The quantity and quality of DNA were assessed using Qubit (Thermo Fisher Scientific Co., Ltd., Shanghai, China) and NanoDrop (Thermo Fisher Scientific Co., Ltd., Shanghai, China), respectively. DNA libraries were prepared using the KAPA Hyper Prep Kit (KAPA Biosystems, Roche, Switzerland) according to the manufacturer’s protocol. Agilent 2100 was used for quality control, and DNA libraries were 75 bp single-end sequenced on Illumina NextSeq 550Dx (Illumina Inc., San Diego, CA, USA).

Raw sequencing data was splited by bcl2fastq, and high-quality sequencing data were generated using Trimmomatic [18] by removing low quality reads, adapter contamination, duplicated and shot (length < 36 bp) reads. Human host sequences were subtracted by mapping to human reference genome (hs37d5) using bowtie2 [19]. Reads that could not be mapped to the human genome were retained and aligned with microorganism genome database for microbial identification by Kraken [20], and for species abundance estimating by Bracken [21]. The microorganism genome database contained genomes or scaffolds of bacteria, fungi, viruses and parasites (download from GenBank release 238, ftp://ftp.ncbi.nlm.nih.gov/genomes/genbank/).

#### Criteria for positive HT-NGS results and clinical assessment

For microbiologic diagnoses, we used the following criteria for positive HT-NGS results. (1) For intracellular bacteria (such as *Mycobacterium tuberculosis complex*, *Nocardia*, and *Legionella pneumophila*), the result was considered positive if a species detected using HT-NGS had a species-specific read number ≥ 1. The threshold for intracellular bacteria was set low just because the difficulty of DNA extraction and low possibility for contamination. (2) For extracellular bacteria (excluding *Mycobacterium tuberculosis complex*, *Nocardia*, and *Legionella pneumophila*), fungi, viruses and parasites, the result was considered positive if a species detected by HT-NGS had at least three non-overlapping reads. (3) A microorganism was always excluded as a routine if it was detected in BALF samples as the same as that in the negative “no-template” control (NTC). However, the results of BALF samples were considered to be positive if the detected reads for a microorganism were ≥ tenfold than that in the NTC. The turnaround time for HT-NGS results were within 24 h after the BALF samples transferred to laboratory, including 14 h for sequencing and 3 h for bioinformatics analysis.

LTx recipients were divided into two groups after clinical assessment: those with pulmonary infections and those without. Pulmonary infection is defined by the presence of one or more of the following symptoms: cough, sputum production, hemoptysis, fever, dyspnea (or increased oxygen requirement) and pleuritic chest pain, plus chest X-ray or CT chest findings of new pulmonary parenchymal disease according to the American Society of Transplantation recommendations for infectious complications in recipients of organ transplantation [22]. For the various microorganisms detected via HT-NGS, screening process for pathogenic pathogens were as follows. First, *Torque teno virus*, *Parvovirus*, intestinal colonization flora (*Citrobacter*, *Roxella*, and *Weissella*), *Staphylococcus epidermidis*, *Ureaplasma*, and anaerobic bacteria which are common but rarely cause pulmonary infections were defined as colonization pathogens base on the patients’ clinical situation. Second, putative pathogens were identified comprehensively by two senior transplant physicians according to the clinical signs, radiological manifestations, conventional microbiological testing results from the recipients, pathogenic microorganisms from the donor (if available) and epidemiology as per the hospital. Third, if the above mentioned two physicians presented consistent analysis results, then causative pathogens were established; in case of disagreement, another senior clinician or clinical microbiologist was drawn into the discussion for determining the causative pathogens.

### Statistical analyses

Data are expressed as “mean ± standard deviation” or “median (interquartile range, IQR)” for continuous variables and “count (percentage)” for categorical variables. McNemar chi-square tests were applied to compare the diagnostic performance of HT-NGS with that of conventional microbiological testing, reported as sensitivity, specificity, positive predictive value (PPV) and negative predictive value (NPV) with their 95% confidence intervals (95%CI). Threshold for statistical significance was set at *P* < 0.05. Statistical analyses were performed, and plots were drawn, using the SPSS statistical software (IBM SPSS Statistics for Windows, Version 21.0. Armonk, NY, USA) and GraphPad Prism software (GraphPad Prism version 6.0.0 for Windows, GraphPad Software, San Diego, CA, USA).

## Results

### Patient characteristics

A total of 51 patients were enrolled in this study, including 40 male and 11 female, with a median age of 60 years (IQR: 53–66 years). Among them, 23 underwent single lung transplantation, 26 underwent bilateral lung transplantation and two underwent heart–lung transplantation. The most prevalent primary indication for LTx was interstitial lung disease (49.02%), followed by chronic obstructive pulmonary disease (31.37%) (Table 1). All LTx recipients received standard triple immunosuppressive regimens consisting of calcineurin inhibitors (tacrolimus), mycophenolate mofetil, and prednisolone. The routine anti-infection regimen was initiated on the day of surgery with carbapenems (meropenem), glycopeptides (vancomycin), triazole (voriconazole) or echinocandins (caspofungin) and ganciclovir to prevent infections by gram-negative bacilli, gram-positive cocci, fungi and CMV respectively.

**Table 1:** Demographic and clinical characteristics of 51 lung transplant recipients

Patient characteristics	Lung transplant recipients, n (%)
Age (IQR, year)	60 (53, 66)
Sex	
Male	40 (78.43%)
Female	11 (21.57%)
BMI (kg/m^2^)	20.82 ± 4.18
Primary disease	
COPD	16 (31.37%)
Interstitial lung disease	25 (49.02%)
Bronchiectasis	5 (9.80%)
Eisenmenger syndrome	2 (3.92%)
Pneumosilicosis	1 (1.96%)
BOS	1 (1.96%)
PLAM	1 (1.96%)
Type of lung transplantation	
Single lung transplantation	23 (45.10%)
Bilateral lung transplantation	26 (50.98%)
Heart–lung transplantation	2 (3.92%)

*COPD* chronic obstructive pulmonary disease, *PLAM* pulmonary lymphangioleiomyomatosis, *BOS* bronchiolitis obliterans syndrome

### Pathogen detection by HT-NGS relative to conventional microbiological testing

**Fig. 2:**
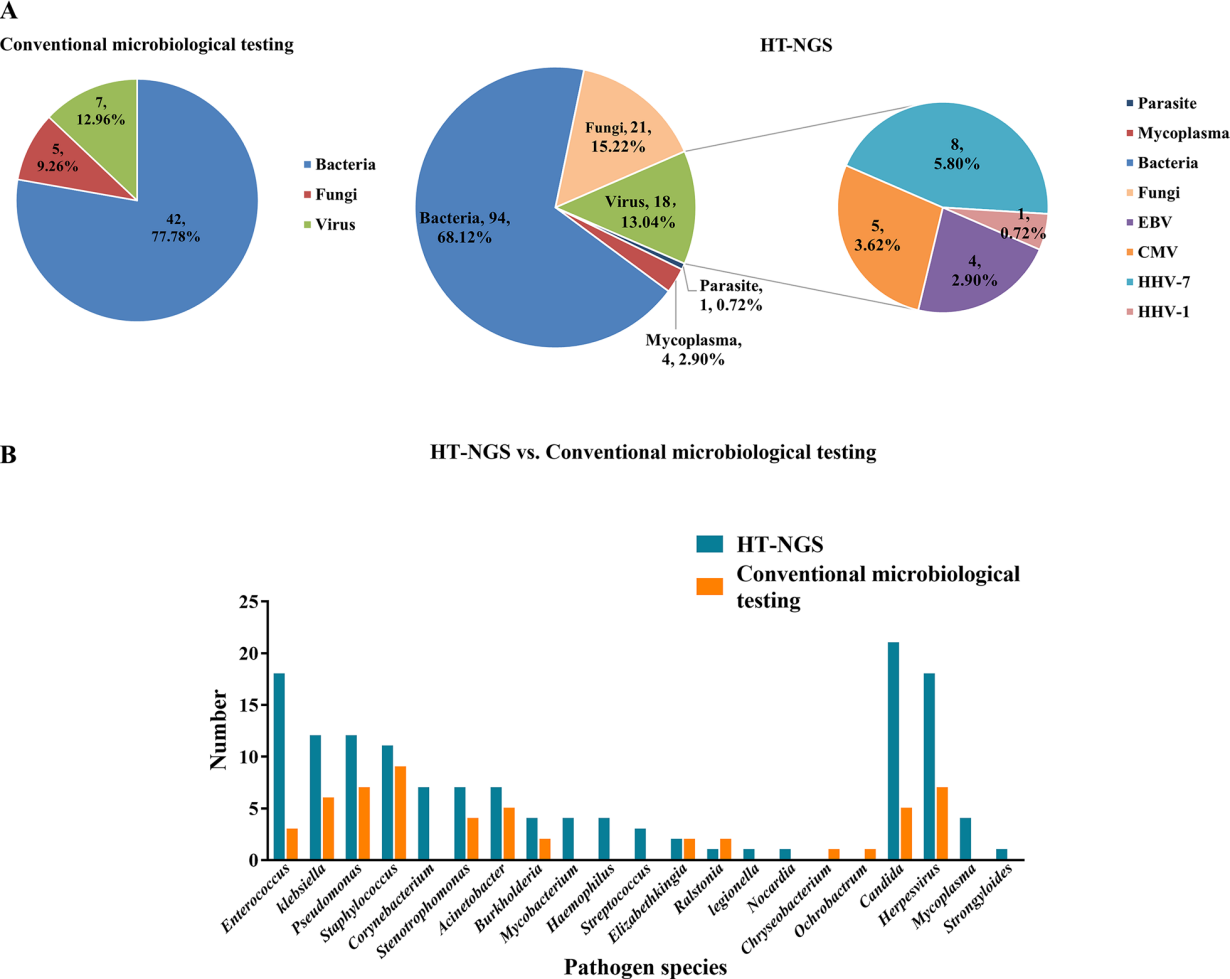
Comparison of pathogens spectrum identified using conventional microbiological testing and high-throughput next-generation sequencing (HT-NGS). **a** Pathogen classification categories identified via conventional microbiological testing and HT-NGS. **b** Pathogen species identified via conventional microbiological testing and HT-NGS

BALF samples were collected from 51 recipients, with a median time of 10 days (IQR: 7–17 days) after lung transplantation. All 51 BALF samples were performed both HT-NGS and conventional microbiological testing. Fifty-four pathogens were detected by conventional microbiological testing from 39 recipients (76.47%), most of which were bacterial (77.78%), fungi (*Candida*) were cultured in five samples (9.26%), and CMV PCR were positive in seven (12.96%) BALF samples. BALF samples from 45 (88.24%) recipients were detected as positive by HT-NGS; among them, 13 (25.49%) were positive for a single pathogen and 32 (62.75%) were positive for two or more pathogens. A total of 138 pathogens were identified by HT-NGS, of which bacteria accounted for 94 (68.12%), and the most prevalent bacteria were *Enterococcus*, *Staphylococcus*, *Pseudomonas*, and *Klebsiella*. All fungi detected by HT-NGS were *Candida* (15.22%), and all viruses were *Herpesvirus* (13.04%). The pathogen spectrum detected by HT-NGS was much wider than that detected by the conventional microbiological testing (Fig. 2)**.** More details of the pathogens detected by HT-NGS and conventional microbiological testing are shown in the Additional file 1: Table S1.

### Comparison of HT-NGS and conventional microbiological testing regarding pathogen detection between pulmonary infection and non-pulmonary infection recipients

All 51 LTx recipients received broad-spectrum antibiotics to prevent infection after the operation. Thirty-five and 16 LTx recipients were assigned to the pulmonary infection and non-pulmonary infection group respectively. Pulmonary infection was diagnosed by a panel of three experienced clinicians after comprehensive analysis of clinical symptoms, signs, laboratory tests, radiologic manifestations, treatment regimen and outcomes according to the criteria mentioned above. Sensitivity and specificity of HT-NGS for diagnosing pulmonary infection were 97.14% and 31.25%, with PPV and NPV of 75.56% and 83.33%, respectively. The sensitivity and specificity of culture for diagnosing pulmonary infection were 68.57% and 62.50%, with PPV and NPV of 80.00% and 47.62%, respectively (Table 2).

**Table 2:** Performance of HT-NGS and conventional microbiological testing in the diagnosis of pulmonary infection in lung transplant recipients

		Infection (35)	Non-infection (16)	Sensitivity% (95%CI)	Specificity% (95%CI)	PPV% (95%CI)	NPV% (95%CI)
HT-NGS	+	34	11	97.14 (83.38–99.85)	31.25 (12.13–58.52)	75.56 (60.14–86.61)	83.33 (36.48–99.12)
	−	1	5				
Conventional microbiological testing	+	24	6	68.57 (50.58–82.57)	62.50 (35.87–83.72)	80.00 (60.87–91.60)	47.62 (26.39–69.66)
	−	11	10				

*HT-NGS* high-throughput next-generation sequencing, *PPV* positive predictive value, *NPV* negative predictive value

### Effect of HT-NGS findings on treatment strategies

Special pathogens, including *Legionella pneumophila*, *Mycobacterium abscessus* and *Strongyloides*, were detected via HT-NGS but not via conventional microbiological testing. Thus, the treatment strategies were amended in the aforementioned three patients as soon as HT-NGS presented positive results (Table 3). The above causative pathogens were detected by HT-NGS and the patients’ condition improved after adjustment of the antibiotic therapy. In addition, antibiotic regimens of the 32 pulmonary infection recipients were continued or adjusted based on the results of HT-NGS with combination of the conventional microbiological testing and drug susceptibility tests. Among them, 16 cases (50.00%) with extensively drug-resistant gram-negative bacilli (including 7 cases of *Pseudomonas aeruginosa*, 5 cases of *Acinetobacter baumannii* and 4 cases of *Klebsiella pneumoniae*) infections were adjusted for antibiotics based on culture and drug susceptibility results. All of the viruses detected via HT-NGS were *Herpesvirus*, although the plasma PCR assay detection was negative during the same period, antiviral prophylaxis with ganciclovir was used routinely. *Candida* detected via HT-NGS was considered to be colonized fungi with a comprehensive evaluation, thus the original antifungal prophylaxis regimen was continued conventionally during early-stage post-lung transplantation.

**Table 3:** High-throughput next-generation sequencing (HT-NGS) results led to change in treatment strategies

Patient	Conventional microbiological testing	HT-NGS results(reads)	Changes in treatment strategies	Outcome
NO. 9	*Candida albicans*	*Strongyloides stercoralis* (5911), *Candida albicans* (122)	Added Ivermectin to the original regimen	Recovery
NO. 23	Negative	*Legionella pneumophila* (16498)	Changed from Piperacillin sodium tazobactam sodium to Azithromycin and Moxifloxacin	Recovery
NO. 30	*Klebsiella pneumoniae*	*Mycobacterium abscessus* (58529), *Klebsiella pneumoniae* (638651), *Enterococcus faecium* (5308)	Added Linezolid and Clarithromycin to the original regimen	Recovery

## Discussion

LTx recipients are at high risk of lifelong infection, and the risk of pulmonary infection is higher in the perioperative period [1]. Fast and accurate pathogen detection is essential for the management of infection in LTx recipients during early-stage post-lung transplantation. Unbiased HT-NGS is a powerful method for pathogen detection, which has been widely used in clinical practice and provides vast quantities of etiological information [23, 24]. However, few studies have comprehensively evaluated the value of HT-NGS in LTx recipients. To the best of our knowledge, this study is the first to investigate the applicability of HT-NGS in pathogen detection in LTx recipients at early-stage post-lung transplantation, in addition to the benefits of amending treatment strategies based on the results of HT-NGS.

We revealed the pathogen spectrum of LTx recipients during the perioperative period using BALF samples which can accurately include the microbes at the level of the alveoli. The results of HT-NGS were similar to those of previous studies, showing that bacteria (68.12%) were the main pathogen in the early-stage post-lung transplantation, and their proportion was much higher than those of fungi (15.22%) and viruses (13.04%) [25, 26]. *Enterococcus*, *Staphylococcus*, *Pseudomonas*, and *Klebsiella* were the most prevalent bacteria. Most of the gram-positive cocci detected via HT-NGS were presented with high reads of gram-negative bacilli and were not considered as pathogenic pathogens. Otherwise, all viruses detected via HT-NGS were *Herpesvirus*, and all fungi were *Candida* which were considered as colonization pathogens. Our findings support the current recommendations for antimicrobial prophylaxis of infections after lung transplantation [27].

As a new technique, HT-NGS is a promising tool for pathogen detection and increasingly being applied in clinical laboratories, but lack of standards and guidelines as a clinical diagnostic protocol nowadays [24]. However, it has the potential to become a promising diagnostic protocol in clinical settings in the near future as emerging evidence shows that HT-NGS had several advantages over the conventional methods in identifying pathogens [12–15]. In addition, our study showed that the positive rate was significantly lower in the results by using conventional culture than that by HT-NGS. The conventional microbiological testing always present false negative results, which was largely attributed to the low sensitivity of the culture, and another reason might be associated with the use of broad-spectrum antimicrobial agents during the postoperative period after LTx in our study. HT-NGS could be used to sequence the entire DNA of a sample, including dead and live pathogens, and its results are less affected by antibiotics [24, 28]. Therefore, it is critical to identify the causative pathogens for an exact anti-infection treatment in clinical settings because the microorganisms detected by HT-NGS might be false positives. In this study, causative pathogens were identified by at least two senior transplant physicians according to the comprehensive clinical and laboratory information, which were described in detail and the criteria were mentioned in the Methods.

Several studies have shown that HT-NGS has higher sensitivity and specificity for the diagnosis of infectious diseases than conventional culture methods [29–31]. Nevertheless, similar to a recent study [32], our study showed that NGS has higher sensitivity (97.14%), but lower specificity (31.25%), when compared to conventional microbiological testing, for the diagnosis of pulmonary infection in LTx recipients. The high false positive rate presented by HT-NGS might be attributed to the following. (1) It has been confirmed that the lungs are not sterile [33], thus it is expected that the microorganisms may contain a variety of non-pathogenic pathogens because of the ultra-high sensitivity of HT-NGS. (2) The donors of LTx recipients in this study were all brain-dead patients who were treated in the intensive care unit and received endotracheal intubation before donation; thus, bacteria colonization might exist in donor lungs. (3) Some pathogens that were killed by broad-spectrum antibiotics during the perioperative period of LTx could still be detected by HT-NGS [7].

In addition, this study showed that HT-NGS had a high NPV (83.33%) in the diagnosis of pulmonary infection, which is very important in the real-world clinical settings. It is known that the perioperative period post-lung transplantation is associated with a very high incidence of both pulmonary infection and acute rejection [1]. However, it is difficult to differentiate infection from rejection at early stage of the episode in clinical settings, as there was no specific manifestations or CT images for any of the complications. Our study results showed that the negative results of the pathogen provided by HT-NGS take a part in helping the clinician to exclude the pulmonary infection, suggesting that the negative result of HT-NGS may have an auxiliary role in the differentiation of pulmonary infection from rejection, but further studies are needed to investigate this point.

In our study, three LTx recipients were found to have special pathogens in the BALF, as detected via HT-NGS, including *Legionella pneumophila*, *Mycobacterium abscessus* and *Strongyloides*, but the conventional microbiological testing results were negative for the same. After adjusting the treatment regimen according to the HT-NGS results, pulmonary infections in these three LTx recipients were controlled and they completely recovered. The results confirm that accurate identification of pathogens plays a crucial role in the selection of antibiotic regimens during the postoperative period after lung transplantation. Our study led to a conclusion similar to those of many previous studies: conventional detection methods have poor ability to detect specific pathogens, while HT-NGS has a higher clinical diagnostic value for rare and difficult-to-culture pathogens [34–37]. HT-NGS can provide more comprehensive etiological evidence, to assist clinicians adjust the treatment regimen in time for the benefit of patients [29, 38]. Therefore, HT-NGS may have broad application prospects in immunocompromised patients, especially in LTx recipients [39].

In addition, among the 35 LTx recipients diagnosed with pulmonary infection, 32 (91.43%) suffered from common pathogenic bacteria, of which 16 (50.00%) were extensively drug-resistant gram-negative bacilli, and the antibiotics needed to be adjusted according to the results of the drug susceptibility tests. However, drug susceptibility tests can only be performed with culturing by the conventional methods at present stage.

Although HT-NGS is a good supplement to current pathogen detection methods, it has limitations. First, unbiased HT-NGS can detect all DNA of pathogens in samples, resulting in higher false positives and lower specificity for diagnosis of pulmonary infection, which may interfere with the clinicians’ judgment. Second, HT-NGS cannot distinguish between contaminant, colonization, and pathogenic pathogens. The positive results should be interpreted by experienced clinicians based on the overall conditions of patients, and should be discussed with microbiologists, if necessary [24]. Third, HT-NGS cannot provide drug susceptibility results for drug-resistant pathogens currently, but antibiotics should be selected according to the results of the traditional culture and drug susceptibility tests in clinics [7].

## Conclusion

HT-NGS has a significantly higher sensitivity to detect pathogens, including rare or difficult-to-culture microbes, which is advantageous compared to the conventional microbiological testing, in terms of detection speed, positive rate, antibiotic intervention and rare pathogen identification. However, HT-NGS has a low specificity and cannot provide drug susceptibility results at present. Therefore, the combination of HT-NGS and conventional microbiological testing can improve the treatment rate and clinical effects, with respect to infections at early-stage post-lung transplantation.

## Limitations

This study has several limitations. First, the sample size was limited, which may have affected the accuracy of the evaluation of HT-NGS performance. Second, this was a cross-sectional study, except for three patients infected with special pathogens, no longitudinal data on how the HT-NGS results were affected by antibiotic therapy. Third, because of the high cost of HT-NGS, we only detected bacteria, fungi, and DNA viruses; therefore, we did not study the possibility of RNA viral etiologies, such as *influenza virus*, *respiratory syncytial virus*, *parainfluenza virus*, and etc., which are prevalent in the infections of the respiratory tract. Finally, although HT-NGS results were discussed among the multidisciplinary group members, some bias may remain due to the potential for false positives and there were no widely accepted quantitative cutoffs for HT-NGS with respect to the diagnosis of causative pathogens.

## Supplementary Information

**Additional file 1: Table S1.** Detailed results of BALF samples detected by HT-NGS and conventional microbiological testing.

## Data Availability

The nonhuman sequence reads from each sample of this study were deposited at NCBI BioProject database (https://www.ncbi.nlm.nih.gov/bioproject) under accession number PRJNA742538.

## Abbreviations

BALF: Bronchoalveolar lavage fluid
BOS: Bronchiolitis obliterans syndrome
CMV: Cytomegaloviruses
COPD: Chronic obstructive pulmonary disease
CT: Computed tomography
EBV: Epstein–Barr virus
GM: Galactomannan
HHV: Human herpesvirus
HT-NGS: High-throughput next-generation sequencing
LTx: Lung transplant
NPV: Negative predictive value
PCR: Polymerase chain reaction
PLAM: Pulmonary lymphangioleiomyomatosis
PPV: Positive predictive value
