# Supplementary material for: High-throughput next-generation sequencing for identifying pathogens during early-stage post-lung transplantation
Source: BMC Pulm Med. 2021 Nov 7;21:348. doi: 10.1186/s12890-021-01723-z (PMC8572506; doi:10.1186/s12890-021-01723-z)
Supplement: Supplementary file 1 — Additional file 1: Table S1. Detailed results of BALF samples detected by HT-NGS and conventional microbiological testing. [file 12890_2021_1723_MOESM1_ESM.docx]

**Additional file 1: Table S1** Detailed results of BALF samples detected by HT-NGS and conventional microbiological testing.

| Patient ID | HT-NGS results (reads) | Smear results | Culture results | CMV-DNA | Other conventional microbiological testing results | Putative pathogens detected by HT-NGS | Causative pathogens |
| --- | --- | --- | --- | --- | --- | --- | --- |
| NO.1 | *Ureaplasma parvum*(1740) | Negative | Negative | Negative | Negative | Negative | Negative |
| NO.2 | *Stenotrophomonas maltophilia*(12) | Negative | *Chryseobacterium indologenes* | Negative | Negative | Negative | Negative |
| NO.3# | *Enterococcus faecium*(11594)*, Enterococcus avium*(276)*, Mycoplasma orale*(7),  *Candida tropicalis*(55),  HHV-7 (516) | Gram-positive cocci | Negative | Negative | Negative | *Enterococcus faecium* (11594)*, Enterococcus avium*(276)*,Candida tropicalis*(55), HHV-7 (516) | *Enterococcus faecium* (11594) |
| NO.4# | *Elizabethkingia anopheles*(8123),  *Enterococcus faecalis*(2416), *Corynebacterium amycolatum*(464), *Mycoplasma hominis*(183), *Staphylococcus aureus*(143), *Klebsiella pneumoniae*(4),  *Candida albicans*(76) | Gram-positive cocci, Gram-negative bacilli | *Elizabethkingia meningosepticum* | Negative | Negative | *Elizabethkingia anopheles*(8123)*  *Enterococcus faecalis*(2416), *Mycoplasma hominis*(183), *Staphylococcus aureus*(143), *Candida albicans*(76) | *Elizabethkingia anopheles*(8123)*  *Enterococcus faecalis*(2416), *Mycoplasma hominis*(183) |
| NO.5 | *Enterococcus casseliflavus*(160), *Haemophilus parahaemolyticus*(5) | Gram-positive cocci | *Stagphylococcus epidermidis* | Negative | Negative | *Enterococcus casseliflavus*(160) | Negative |
| NO.6# | *Stenotrophomonas maltophilia*(2508),  *Mycoplasma hominis*(1914), *Klebsiella pneumoniae*(983),  *Haemophilus parainfluenzae*(92),  *Enterococcus faecalis*(13),  *Candida albicans*(22) | Gram-negative bacilli | *Klebsiella pneumoniae* | Positive | Negative | *Stenotrophomonas maltophilia*(2508), *Mycoplasma hominis*(1914), *Klebsiella pneumoniae*(983) **Haemophilus parainfluenzae*(92), *Candida albicans*(22) | *Klebsiella pneumoniae*(983), *Mycoplasma hominis*(1914) |
| NO.7 | *Leuconostoc garlicum*(134), *Haemophilus parahaemolyticus*(17), *Staphylococcus aureus*(15), *Corynebacterium striatum*(5), *Haemophilus parainfluenzae*(3),  HHV-7(8) | Negative | Negative | Negative | Negative | HHV-7(8) | Negative |
| NO.8# | *Pseudomonas aeruginosa*(4358), *Haemophilus influenzae*(3782), *Enterococcus faecalis*(2129), *Corynebacterium resistens*(416), *Klebsiella pneumoniae*(201), *Staphylococcus aureus*(15), *Streptococcus pneumoniae*(5),  HHV-7(512) | Gram-negative bacilli,  Gram-positive cocci | *Pseudomonas aeruginosa* | Negative | Negative | *Pseudomonas aeruginosa*(4358)*  *Enterococcus faecalis*(2129), *Haemophilus influenzae*(3782), *Enterococcus faecalis*(2129), *Corynebacterium resistens*(416),  *Klebsiella pneumoniae*(201), HHV-7(512) | *Pseudomonas aeruginosa*(4358)*  *Enterococcus faecalis*(2129), *Klebsiella pneumoniae*(201) |
| NO.9# | *Strongyloides stercoralis*(5911), *Candida albicans*(122) | Negative | *Candida albicans* | Negative | Negative | *Strongyloides stercoralis* (5911),  *Candida albicans* (122)* | *Strongyloides stercoralis* (5911) |
| NO.10# | *Stenotrophomonas maltophilia*(4738), *Corynebacterium resistens*(1450), *Klebsiella pneumoniae*(27),  HHV-7(716),  CMV(55) | Gram-negative bacilli | *Klebsiella pneumoniae* | Negative | Negative | *Stenotrophomonas maltophilia*(4738), *Corynebacterium resistens*(1450), HHV-7(716), CMV(55),  *Klebsiella pneumoniae*(27)* | *Klebsiella pneumoniae*(27)* |
| NO.11# | *Enterococcus avium*(4999), *Staphylococcus aureus*(29), *Cronobacter sakazakii*(10), *Enterococcus faecium*(5),  *Rothia aeria*(4),  *Acinetobacter baumannii*(4) | Gram-positive cocci | *Enterococcus avium* | Negative | Negative | *Enterococcus avium*(4999)* | *Enterococcus avium*(4999)* |
| NO.12 | *Corynebacterium striatum*(3811) | Negative | *Staphylococcus haemolyticus* | Negative | Negative | *Corynebacterium striatum*(3811) | Negative |
| NO.13# | *Acinetobacter baumannii*(64),  *Finegoldia magna*(8),  *Mycobacterium avium*(3),  *Human parvovirus*(58),  *Nocardia farcinica*(3) | Gram-negative bacilli | *Stenotrophomonas maltophilia* | Negative | Negative | *Acinetobacter baumannii*(64), *Mycobacterium avium*(3), *Nocardia farcinica*(3) | *Acinetobacter baumannii*(64) |
| NO.14# | *Pseudomonas aeruginosa*(52314), *Acinetobacter baumannii*(1693), *Klebsiella pneumoniae*(500) | Gram-negative bacilli | *Acinetobacter baumannii* | Negative | Negative | *Pseudomonas aeruginosa*(52314)*,  *Acinetobacter baumannii*(1693), *Klebsiella pneumoniae*(500) | *Pseudomonas aeruginosa*(52314)*,  *Acinetobacter baumannii*(1693), *Klebsiella pneumoniae*(500) |
| NO.15# | *Acinetobacter baumannii*(324163), *Klebsiella pneumoniae*(15625), *Pseudomonas aeruginosa*(7447), *Enterococcus faecium*(876), *Corynebacterium striatum*(137), *Haemophilus parainfluenzae*(43),  EBV (4),  *Candida parapsilosis*(26)  EBV(44) | Gram-negative bacilli | *Acinetobacter baumannii, Klebsiella pneumoniae, Pseudomonas aeruginosa* | Positive | Negative | *Acinetobacter baumannii*(324163)*, *Klebsiella pneumoniae*(15625)*, *Pseudomonas aeruginosa*(7447)*,  *Enterococcus faecium*(876), *Corynebacterium striatum*(137), *Candida parapsilosis*(26), EBV(44) | *Acinetobacter baumannii*(324163)*, *Klebsiella pneumoniae*(15625)*, *Pseudomonas aeruginosa*(7447)* |
| NO.16# | *Klebsiella pneumoniae*(1334), *Pseudomonas aeruginosa*(21), *Enterococcus faecalis*(62) | Gram-negative bacilli,  Gram-positive cocci | Staphylococcus haemolyticus | Negative | Negative | *Klebsiella pneumoniae*(1334), *Pseudomonas aeruginosa*(21), *Enterococcus faecalis*(62) | *Klebsiella pneumoniae*(1334), *Pseudomonas aeruginosa*(21) |
| NO.17 | *Staphylococcus aureus*(515), *Streptococcus pneumoniae*(432),  *Haemophilus parahaemolyticus*(16), | Negative | Negative | Negative | Negative | *Staphylococcus aureus*(515), *Streptococcus pneumoniae*(432) | Negative |
| NO.18# | *Acinetobacter baumannii*(10337), *Achromobacter xylosoxidans*(4120), *Enterococcus faecalis*(1790), *Haemophilus parahaemolyticus*(183), *Streptococcus pneumoniae*(182), *Staphylococcus aureus*(124), *Streptococcus pneumoniae*(33),  *Candida haemulonii*(22),  *Pichia kudriavzevii*(4)  HHV-7(7) | Gram-negative bacilli,  Gram-positive cocci | Negative | Negative | Negative | *Acinetobacter baumannii*(10337), *Enterococcus faecalis*(1790), *Streptococcus*, *parahaemolyticus*(183), *pneumoniae*(182), *Staphylococcus aureus*(124), *Candida haemulonii*(22) | *Acinetobacter baumannii*(10337), *Enterococcus faecalis*(1790) |
| NO.19# | *Ureaplasma urealyticum*(4098), *Enterococcus faecium*(396), *Klebsiella pneumoniae*(16),  CMV(114) | Gram-positive cocci | *Staphylococcus haemolyticus* | Positive | Negative | *Enterococcus faecium*(396), *Klebsiella pneumoniae*(16),  CMV(114) | *Enterococcus faecium*(396), *Klebsiella pneumoniae*(16) |
| NO.20# | *Ureaplasma urealyticum*(2164), *Ureaplasma parvum*(66), *Haemophilus parahaemolyticus*(13)，*Streptococcus pneumoniae*(7), *Streptococcus pneumoniae*(4),  *Corynebacterium striatum*(3) | Negative | *Staphylococcus capitis* | Positive | Negative | Negative |  |
| NO.21# | *Pseudomonas aeruginosa*(7656), *Stenotrophomonas maltophilia*(5142),  *Staphylococcus aureus*(18), *Enterococcus raffinosus*(12),  *Candida albicans*(93) | Gram-negative bacilli | *Pseudomonas aeruginosa* | Negative | Negative | *Pseudomonas aeruginosa*(7656)*,  *Stenotrophomonas maltophilia*(5142), *Candida albicans*(93) | *Pseudomonas aeruginosa*(7656)* |
| NO.22# | *Acinetobacter baumannii*(1653141),  *Enterococcus faecalis*(80) | Gram-negative bacilli | *Acinetobacter baumannii* | Negative | Negative | *Acinetobacter baumannii*(1653141)*, *Enterococcus faecalis*(80) | *Acinetobacter baumannii*(1653141)* |
| NO.23# | *Legionella pneumophila*(16498) | Gram-negative bacilli | Negative | Negative | Negative | *Legionella pneumophila*(16498) | *Legionella pneumophila*(16498) |
| NO.24# | *Stenotrophomonas maltophilia*(495029),  *Staphylococcus haemolyticus*(7982),  *Corynebacterium striatum*(1477),  *Tropheryma whipplei*(46),  *Finegoldia magna*(44), *Staphylococcus aureus*(23), *Enterococcus faecium*(22), *Escherichia coli* (10),  *Mycobacterium abscessus*(8),  *Candida parapsilosis*(122),  HHV-1(438) | Gram-negative bacilli | *Stenotrophomonas maltophilia, Staphylococcus haemolyticus* | Negative | Negative | *Stenotrophomonas maltophilia*(495029)*, *Staphylococcus haemolyticus*(7982),  *Corynebacterium striatum*(1477), *Mycobacterium abscessus*(8),  *Candida parapsilosis*(122),  HHV-1(438) | *Stenotrophomonas maltophilia*(495029)* |
| NO.25 | *Corynebacterium striatum*(1576),  *Corynebacterium resistens*(22) | Negative | Negative | Negative | Negative | Negative | Negative |
| NO.26 | *Weissella confuse*(211),  *Morganella morganii*(27), *Bacteroides fragilis*(23), *Corynebacterium striatum*(22), *Streptococcus pneumoniae*(3) | Gram-negative bacilli | Acinetobacter baumannii | Negative | Negative | Negative | Negative |
| NO.27# | *Elisabella anopheles*(2440584), *Stenotrophomonas* *maltophilia*(1290214), *Enterococcus* *faecium*(66342), *Rothia* *aeria*(422),  *Haemophilus parainfluenzae*(37), *Streptococcus pneumoniae*(30),  *Candida albicans*(34) | Gram-negative bacilli,  Gram-positive cocci | *Elizabethkingia meningosepticum* | Negative | Negative | *Elisabella anopheles*(2440584)*, *Stenotrophomonas* *maltophilia*(1290214), *Enterococcus* *faecium*(66342), *Candida albicans*(34) | *Elisabella anopheles*(2440584)*, *Stenotrophomonas* *maltophilia*(1290214), *Enterococcus* *faecium*(66342) |
| NO.28# | *Burkholderia multivorans*(1721), *Citrobacter koseri*(210), *Staphylococcus aureus*(69), *Clostridium perfringens*(13), *Enterococcus faecalis*(11), *Haemophilus parainfluenzae*(10) | Gram-negative bacilli | Negative | Negative | Negative | *Burkholderia multivorans*(1721), *Staphylococcus aureus*(69) | *Burkholderia multivorans*(1721), *Staphylococcus aureus*(69) |
| NO.29# | *Pseudomonas aeruginosa*(11366), *Streptococcus pneumoniae*(76),  *Candida dubliniensis*(43),  *Candida albicans*(6),  *Candida glabrata*(5),  HHV-7(317) | Gram-negative bacilli,  Gram-positive cocci | *Pseudomonas aeruginosa* | Negative | Negative | *Pseudomonas aeruginosa*(11366)*, *Streptococcus pneumoniae*(76),  *Candida dubliniensis*(43),  *Candida albicans*(6),  *Candida glabrata*(5),  HHV-7(317) | *Pseudomonas aeruginosa*(11366)* |
| NO.30# | *Mycobacterium abscessus* (58529), *Klebsiella pneumoniae* (638651), *Enterococcus faecium* (5308) | Gram-negative bacilli,  Gram-positive cocci | *Klebsiella pneumoniae* | Negative | GM test (+) | *Klebsiella pneumoniae*(638651)*, *Mycobacterium abscessus* (58529), *Enterococcus faecium* (5308) | *Klebsiella pneumoniae*(638651)*, *Mycobacterium abscessus* (58529), *Enterococcus faecium* (5308) |
| NO.31# | *Klebsiella pneumoniae*(40258),  *Stenotrophomonas maltophilia*(11125),  *Klebsiella oxytoca*(4569),  *Acinetobacter baumannii*(454),  *Burkholderia cepacian*(17)， | Gram-negative bacilli | *Klebsiella pneumoniae* | Negative | GM test (+) | *Klebsiella pneumoniae*(40258)*，*Stenotrophomonas maltophilia*(11125)*,  *Klebsiella oxytoca*(4569), *Acinetobacter baumannii*(454)*,  *Candida albicans*(91)，*Burkholderia cepacian*(17) | *Klebsiella pneumoniae*(40258)* |
| NO.32 | *Enterococcus faecium*(8),  EBV(73) | Negative | *Stenotrophomonas maltophilia* | Negative | GM test (+) | EBV(73) | Negative |
| NO.33 | *Corynebacterium striatum*(1269), *Corynebacterium resistens*(592), *Klebsiella aerogenes*(8) | Gram-negative bacilli | Negative | Negative | Negative | *Corynebacterium striatum*(1269) | Negative |
| NO.34# | *Stenotrophomonas* *maltophilia*(62365),  *Haemophilus parainfluenzae*(109), *Pseudomonas aeruginosa*(69), *Staphylococcus aureus*(52), *Finegoldia magna*(5),  *Candida albicans*(161) | Gram-negative bacilli | *Stenotrophomonas maltophilia* | Negative | Negative | *Stenotrophomonas* *maltophilia*(62365)*, *Haemophilus parainfluenzae*(109), *Pseudomonas aeruginosa*(69), *Staphylococcus aureus*(52), *Candida albicans*(161) | *Stenotrophomonas* *maltophilia*(62365)*, *Pseudomonas aeruginosa*(69) |
| NO.35 | *Staphylococcus haemolyticus*(11327),  *Haemophilus parainfluenzae*(5), *Mycobacterium abscessus*(6) | Gram-negative bacilli,  Gram-positive cocci | *Staphylococcus haemolyticus* | Negative | Negative | *Staphylococcus haemolyticus*(11327)*, *Mycobacterium abscessus*(6) | Negative |
| NO.36# | *Staphylococcus haemolyticus*(37541)  *Staphylococcus aureus*(50) | Gram-negative bacilli,  Gram-positive cocci | *Staphylococcus haemolyticus, Klebsiella pneumoniae* | Negative | Negative | *Staphylococcus haemolyticus*(37541)*  *, Staphylococcus aureus*(50) | *Staphylococcus haemolyticus*(37541)**, Klebsiella pneumoniae* |
| NO.37 | *Mycoplasma hominis*(51), *Haemophilus parainfluenzae*(32), *Staphylococcus aureus*(3) | Negative | Negative | Negative | Negative | *Mycoplasma hominis*(51) | Negative |
| NO.38# | *Staphylococcus haemolyticus*(608)  *Enterococcus faecium*(59),  EBV(55) | Gram-positive cocci | *Staphylococcus haemolyticus, Enterococcus faecium* | Negative | Negative | *Staphylococcus haemolyticus*(608)*  *Enterococcus faecium*(59)*, EBV(55) | *Staphylococcus haemolyticus*(608)*  *Enterococcus faecium*(59)* |
| NO.39# | *Pseudomonas aeruginosa*(114204),  *Enterococcus avium*(21346),  *Acinetobacter baumannii*(8),  *Torque teno virus*(57) | Gram-negative bacilli,  Gram-positive cocci | *Ralstonia mannitolilytica, Ochrobactrum anthropi* | Negative | GM test (+) | *Pseudomonas aeruginosa*(114204),  *Enterococcus avium*(21346) | *Pseudomonas aeruginosa*(114204),  *Enterococcus avium*(21346) |
| NO.40# | *Klebsiella pneumoniae*(31)，  *Candida albicans*(749)，  HHV-7(79),  CMV(12) | Gram-negative bacilli | *Candida albicans* | Positive | Negative | *Candida albicans*(749)*, *Klebsiella pneumoniae*(31), HHV-7(79),  CMV(12) | *Candida albicans*(749)*, *Klebsiella pneumoniae*(31) |
| NO.41# | *Pseudomonas aeruginosa*(3145),  *Klebsiella pneumoniae*(442),  *Ralstonia mannitolilytica*(105),  CMV(83),  HHV-7(72) | Gram-negative bacilli | *Ralstonia mannitolilytica* | Negative | Negative | *Ralstonia mannitolilytica*(105)*, *Pseudomonas aeruginosa*(3145), *Klebsiella pneumoniae*(442), CMV(83), HHV-7(72) | *Pseudomonas aeruginosa*(3145), *Klebsiella pneumoniae*(442) |
| NO.42 | *Staphylococcus aureus*(8), *Haemophilus parainfluenzae*(4), *Candida parapsilosis*(1599),  *Candida albicans*(4) | Negative | *Candida parapsilosis* | Negative | Negative | *Candida parapsilosis*(1599)* | Negative |
| NO.43# | *Pseudomonas aeruginosa*(729773), *Enterococcus avium*(35738), *Stenotrophomonas maltophilia*(1663),  *Ureaplasma urealyticum*(876), *Corynebacterium striatum*(11), *Staphylococcus aureus*(4),  *Candida parapsilosis*(4) | Gram-negative bacilli,  Gram-positive cocci | *Pseudomonas aeruginosa* | Negative | Negative | *Pseudomonas aeruginosa*(729773)*, *Enterococcus avium*(35738), *Stenotrophomonas maltophilia*(1663), *Candida parapsilosis*(4) | *Pseudomonas aeruginosa*(729773)*, *Enterococcus avium*(35738) |
| NO.44 | *Staphylococcus aureus* (26), *Corynebacterium striatum*(6),  CMV(26) | Negative | Negative | Positive | Negative | CMV(26) | Negative |
| NO.45# | *Enterococcus faecium*(15735), *Burkholderia multivorans* (10786), *Leuconostoc mesenteroides*(76),  *Candida glabrata*(37),  *Candida albicans*(5) | Gram-negative bacilli,  Gram-positive cocci | *Burkholderia multivorans* | Positive | Negative | *Burkholderia multivorans* (10786)*, *Enterococcus faecium*(15735), *Candida glabrata*(37),  *Candida albicans*(5) | *Burkholderia multivorans* (10786)*, *Enterococcus faecium*(15735) |
| NO.46 | *Staphylococcus aureus* (139),  *mycoplasma hominis*(92) | Gram-positive cocci | *Enterococcus faecium* | Negative | Negative | *Staphylococcus aureus* (139),  *mycoplasma hominis*(92) | Negative |
| NO.47# | *Acinetobacter baumannii*(1135642) | Gram-negative bacilli | *Acinetobacter baumannii* | Negative | Negative | *Acinetobacter baumannii*(1135642)* | *Acinetobacter baumannii*(1135642)* |
| NO.48# | *Pseudomonas aeruginosa*(12911) | Gram-negative bacilli | *Pseudomonas aeruginosa, Candida albicans* | Negative | GM test (+) | *Pseudomonas aeruginosa*(12911)* | *Pseudomonas aeruginosa*(12911)* |
| NO.49# | *Burkholderia multivorans* (2422),  *Torque teno virus* (100) | Gram-negative bacilli | *Burkholderia multivorans* | Negative | GM test (+) | *Burkholderia multivorans* (2422)* | *Burkholderia multivorans* (2422)* |
| NO.50# | *Pseudomonas aeruginosa*(44062),  *Enterococcus faecium*(395),  *Citrobacter koseri* (513),  Candida tropicalis(14),  *Torque teno virus*(74),  EBV(24), HHV-7(3) | Gram-negative bacilli,  Gram-positive cocci | *Pseudomonas aeruginosa, Candida tropicalis* | Negative | Negative | *Pseudomonas aeruginosa*(44062)*, *Enterococcus faecium*(395), Candida tropicalis(14), EBV(24) | *Pseudomonas aeruginosa*(44062)* |
| NO.51 | Negative | Negative | Negative | Negative | Negative | Negative | Negative |

#: Infection patient; *: Pathogen detected by HT-NGS were validated; Other conventional microbiological testing results including GM test, *Mycobacterium tuberculosis* DNA testing, and Xpert MTB/RIF assay; BALF, bronchoalveolar lavage fluid; CMV, *cytomegalovirus*; EBV, *Epstein-Barr virus*; HHV-7*, Human herpesvirus* 7; HT-NGS, high-throughput next-generation sequencing.
